# Case Report: Co‐Occurrence of Lung Adenocarcinoma and Congenital Dysfibrinogenemia—Diagnostic and Perioperative Management Challenges

**DOI:** 10.1002/cnr2.70512

**Published:** 2026-03-06

**Authors:** He Zheng, Qingsong Wang, Mingpu Wang, Shu Luo, Yuzhen Ma, Zhengqiang Wan

**Affiliations:** ^1^ Department of Thoracic Surgery Suining First People's Hospital Suining Sichuan China; ^2^ Oncology Suining First People's Hospital Suining Sichuan China; ^3^ Department of Oral and Maxillofacial Surgery Suining First People's Hospital Suining Sichuan China

**Keywords:** congenital dysfibrinogenemia, fibrinogen replacement therapy, lung adenocarcinoma, minimally invasive thoracic surgery, perioperative coagulation management

## Abstract

**Background:**

Congenital dysfibrinogenemia (CD), a rare autosomal dominant coagulation disorder, poses significant perioperative challenges in oncologic surgery due to hypofibrinogenemia and variable bleeding‐thrombosis risks.

**Case:**

A 67‐year‐old woman presented with a 2.9 × 1.4 cm spiculated mass in the right middle lobe (RML) and persistent hypofibrinogenemia (0.56–0.58 g/L). Despite conventional fresh frozen plasma and cryoprecipitate transfusions, fibrinogen levels remained critically low. Preoperative optimization with human fibrinogen concentrates (total 4.0 g) normalized levels to 1.76 g/L within 24 h, enabling video‐assisted thoracoscopic (VATS) RML lobectomy with systematic lymphadenectomy (pT1cN0M0, Stage IA3). Postoperatively, fibrinogen gradually declined to 0.68 g/L at 1‐month follow‐up, necessitating extended surveillance. Genetic testing identified a heterozygous FGA c.103C>T (Arg35Cys) mutation, confirming CD diagnosis and thrombotic risk.

**Conclusion:**

This case establishes three paradigms: (1) Mandatory comprehensive coagulation profiling in pulmonary resection candidates to identify CD; (2) superiority of fibrinogen concentrate over plasma‐derived products for rapid, sustained hemostasis in CD; (3) individualized thromboprophylaxis balancing hemorrhagic‐thrombotic risks through genetic risk stratification.

## 1 Introduction

Lung cancer is the leading cause of cancer incidence and mortality in men globally, and the third most common cancer as well as the second leading cause of cancer death in women [1]. Lung adenocarcinoma (LUAC)—the most prevalent subtype of non‐small cell lung cancer (NSCLC), accounting for ~60% of cases—has a large patient cohort and the highest mortality among all lung cancer deaths [2], thus, its treatment is a cornerstone of lung cancer management. Currently, the overall prognosis of LUAC patients remains poor, with a 5‐year overall survival (OS) of only 15% [3]. CD, an autosomal dominant genetic disorder, stems from fibrinogen (Fg) gene defects that disrupt Fg structure and function; most cases involve heterozygous mutations [4]. Clinically, CD is rare and phenotypically heterogeneous: most patients are asymptomatic; 25% experience bleeding; 20% have thrombotic events, some present with both, and others develop recurrent miscarriage or poor wound healing [5]. CD patients typically exhibit a mild bleeding phenotype with minimal, inconsequential bleeding—however, vigilance is warranted in women of childbearing age, surgical candidates, or those with severe trauma. Close monitoring and timely intervention are critical to prevent intractable bleeding [6].

While CD is well‐documented, coexistence with LUAC is exceedingly rare, and perioperative management remains poorly characterized. This study reports a novel case of concurrent LUAC and CD in a 67‐year‐old woman, emphasizing three unique features: (1) Unprecedented perioperative challenges: The patient had hypofibrinogenemia unresponsive to fresh frozen plasma (FFP) or cryoprecipitate, but human fibrinogen concentrates (HFC) effectively corrected it. (2) Genetic and familial insights: Genetic testing identified a heterozygous FGA mutation (c.103C>T), and pedigree analysis confirmed familial CD inheritance. This sheds light on CD genetic basis and its relevance to surgical patients. (3) Multidisciplinary management: The case demonstrates successful preoperative CD diagnosis and hemostatic optimization via a multidisciplinary team (MDT), reducing surgical risks.

## 2 Case Presentation

### 2.1 Clinical History and Diagnostic Workup

**FIGURE 1:**
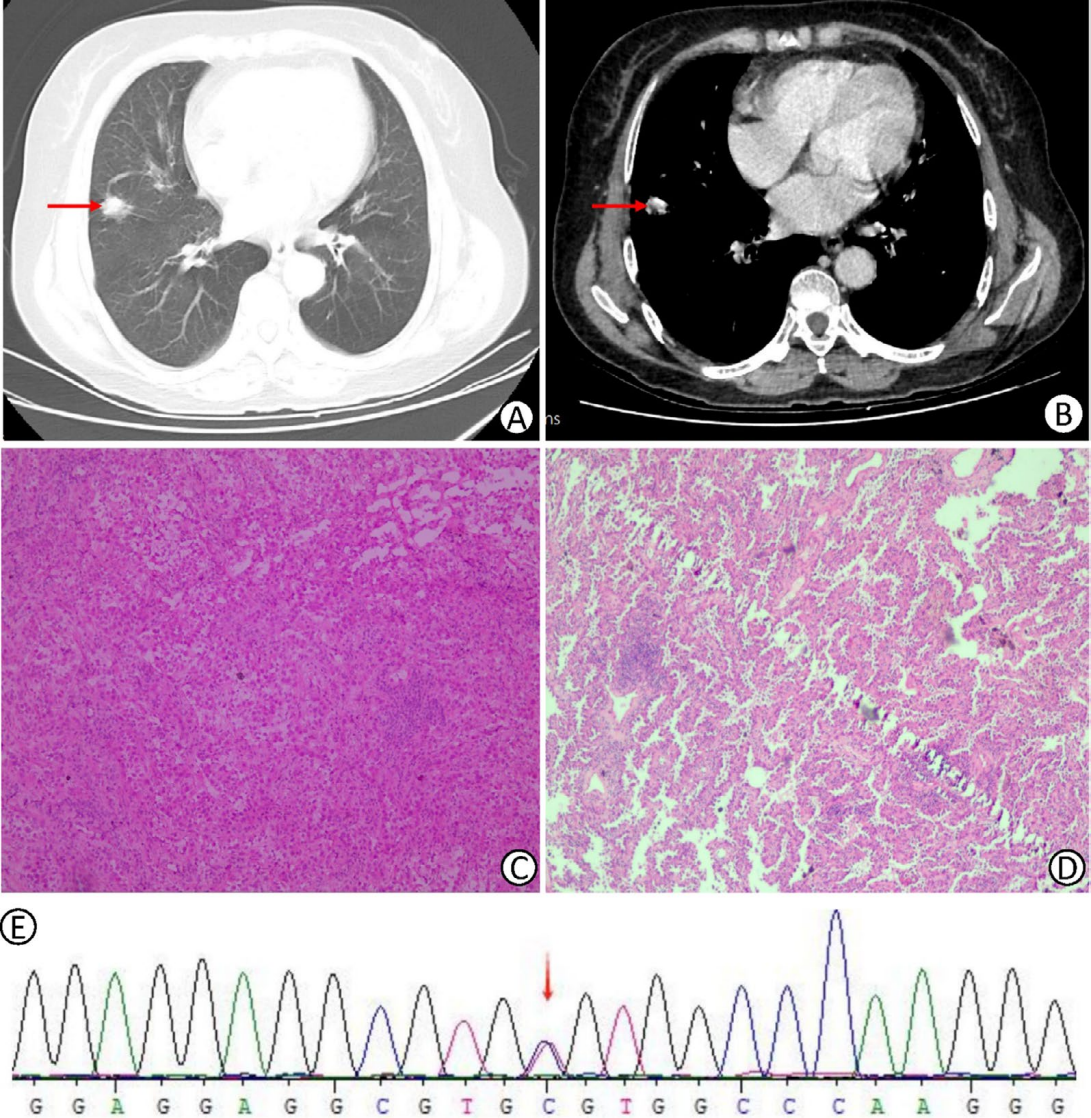
(A) Chest CT suggesting a nodule in the upper lobe of the right lung (indicated by the red arrow). (B) Enhanced CT of the chest suggests a nodule in the upper lobe of the right lung (indicated by the red arrow). (C) Intraoperative rapid pathologic findings suggesting lung adenocarcinoma. TTF1 (+), NapsinA (+), CK7 (+), EGFR (weak), ALK (+), EGFR (weak), EGFR (+), ALK (weak), EGFR (+), ALK (+). (+), ALK (−), ki67 positivity was about 3%. (D) Postoperative pathologic findings suggesting lung adenocarcinoma. 90% of adenocarcinoma with ependymal growth, 10% of adenoids, localized metastasis in the end of the Limba node, respectively: Group 4 (0/1), Group 7 (0/1), Group 8 (0/1), Group 9 (0/1), bronchial margins and pleura did not show any cancerous involvement. (E) Sequencing map of the patient's fibrinogen alpha chain gene. Whole‐exome sequencing results suggested heterozygous mutation of c.103C>T (arg35Cys) in the FGA gene (mutation sites indicated by red arrows).

A 67‐year‐old non‐smoking female was admitted to the Department of Thoracic Surgery at Suining First People's Hospital for diagnosis and treatment on February 19, 2024. Her chief complaint included a 4‐year history of incidentally detected pulmonary nodules and recent‐onset chest pain. Chest CT showed a solid nodule (2.9 × 1.4 cm) in the right middle lobe, classified as Lung‐RADS 4B (Figure 1A,B). Preoperative coagulation tests revealed severe hypofibrinogenemia (Clauss method) (0.56 g/L), and the fibrinogen antigen assay was not performed. Despite transfusions of FFP and cryoprecipitate, fibrinogen levels remained low (0.58 g/L), whereas human fibrinogen infusion normalized coagulation (Table 1).

**TABLE 1:** Changes in coagulation in patients after admission to the hospital.

Date	Intervention	Coagulation function				
		PT (s) (11–15)^a^	APTT (s) (25–37)	Fibrinogen (g/L) (2–4)	TT (S) (12–16)	D‐D dimer (mg/L) (0–0.5)
2024‐2‐19	—	15.30	36.10	0.56	33.10	0.21
2024‐2‐20	—	15.40	36.40	0.58	29.90	0.31
2024‐2‐22	Infusion of cold precipitated 10 U	14.70	36.50	0.69	29.50	0.08
2024‐2‐23	Infusion of fresh frozen plasma 400 mL	15.10	29.50	0.80	26.40	1.47
2024‐2‐25	—	15.00	34.50	0.67	31.70	0.23
2024‐2‐28	Infusion of human fibrinogen 2.0 g	14.10	34.80	1.19	23.40	0.38
2024‐2‐29	Infusion of human fibrinogen 2.0 g	13.80	37.50	1.76	23.00	0.39

*Note:* —: No special treatment.

Abbreviations: APTT: activated partial thromboplastin time; PT: prothrombin time.

^a^ Normal reference range.

### 2.2 Surgical Intervention and Pathological Findings

The patient underwent thoracoscopic right middle lobectomy with lymph node dissection. Intraoperative frozen section and postoperative pathology confirmed LUAC (Figure 1C,D). Immunohistochemistry showed TTF1(+), NapsinA(+), and CK7(+). Genetic analysis identified a heterozygous FGA mutation (c.103C>T, p.Arg35Cys) (Figure 1E), consistent with CD. Staging was pT1cN0M0 (IA3).

### 2.3 Family History and Pedigree Analysis

**FIGURE 2:**
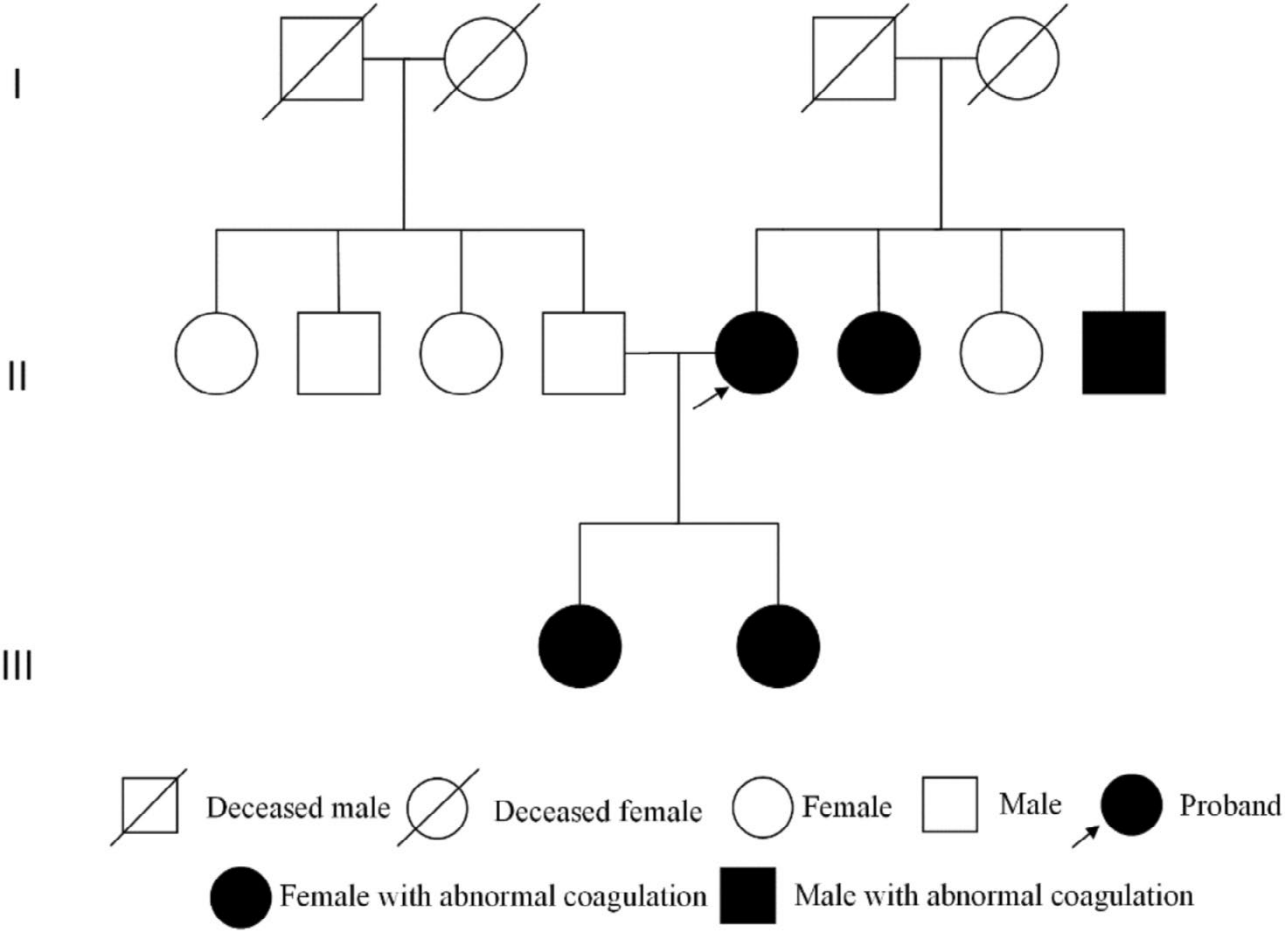
Pedigree study of patients with CD.

Given the CD suspicion, family history revealed multiple relatives with coagulation abnormalities. Pedigree analysis indicated an autosomal dominant pattern, supporting hereditary CD (Figure 2).

### 2.4 Perioperative Management

1. Bleeding Prevention: Chest drainage was monitored closely (Table 2); no hemorrhagic events occurred.
2. Coagulation Monitoring: Postoperative fibrinogen declined gradually (Table 3), but no intervention was needed.
3. Outcome: At 1‐month follow‐up, fibrinogen was 0.68 g/L, with no bleeding, thrombosis, or tumor recurrence.

**TABLE 2:** Postoperative chest drains drainage statistics.

Date	Right chest drains drainage (mL)	Color of drainage fluid
2024‐3‐1	120	Dark red bloody fluid
2024‐3‐2	400	Reddish bloody fluid
2024‐3‐3	160	Reddish bloody fluid
2024‐3‐5	200	Yellowish
2024‐3‐6	120	Yellowish

**TABLE 3:** Postoperative coagulation changes.

Date	Coagulation function				
	PT (s) (11–15)	APTT (s) (25–37)	Fibrinogen (g/L) (2–4)	TT (s) (12–16)	D‐D dimer (mg/L) (0–0.5)
2024‐3‐1	13.80	35.90	1.59	24.40	1.75
2024‐3‐2	15.00	38.40	1.50	23.40	1.53
2024‐3‐3	15.80	42.20	1.52	27.00	1.27
2024‐3‐5	14.30	38.50	1.49	29.50	2.00
2024‐3‐8	15.30	36.40	0.98	33.70	3.44

## 3 Discussion

According to the 2023 global cancer statistics, lung cancer remains the most common malignant neoplasm of the lung worldwide [7]. Since 2004, LUAC has emerged as the predominant histological subtype of lung cancer globally [8]. Pathologically, LUAC is characterized by large cells, nuclear atypia, and abundant cytoplasm. Compared to small cell lung cancer (SCLC), NSCLC exhibits slower growth, division, and later metastatic spread [9]. Factors influencing the occurrence and progression of LUAC include air pollution, secondhand smoke, occupational carcinogens, as well as genetic and gene alterations [10, 11].

Lobectomy combined with systematic lymph node dissection was once the standard surgical approach for lung cancer. The guiding principles of this surgery are complete resection of the primary pulmonary tumor, thorough dissection of hilar and mediastinal lymph nodes, and maximal preservation of functional lung tissue. This strategy aims to retain pulmonary function to the greatest extent, ensuring patients' normal postoperative activity and quality of life [12]. The surgical procedure performed in this case was VATS right middle lobectomy with lymph node dissection.

CD is primarily characterized by structural abnormalities in fibrinogen that impair its functional properties [13], manifesting as abnormal release of fibrinopeptide A/B, impaired fibrin monomer polymerization, defective fibrin cross‐linking, and dysregulated fibrinolysis [14]. According to the guidelines recommended by *Diagnosis and classification of congenital fibrinogen disorders: communication from the SSC of the ISTH* [15], a stepwise approach is suggested for the confirmation of diagnosis of CD (congenital dysfibrinogenemia): the first step involves routine clotting time tests (e.g., activated partial thromboplastin time (APTT) and prothrombin time (PT) and fibrinogen assays; the second step is to complete genetic typing). Unfortunately, this patient was diagnosed via genetic testing and thus did not undergo this assay. Clinical manifestations of hereditary dysfibrinogenemia are highly heterogeneous, ranging from asymptomatic presentation to bleeding, thrombosis, or a combination. Bleeding symptoms include epistaxis, easy bruising, menorrhagia, hematomas (subcutaneous or joint), postoperative hemorrhage, antepartum/postpartum bleeding, and delayed wound healing—most are mild to moderate. Thrombotic events involve lower extremity deep vein thrombosis, thrombophlebitis, pulmonary embolism, arterial thrombosis, or combined arterial/venous thrombosis [16]. This patient was asymptomatic preoperatively, with no prior bleeding or thrombotic events, and was diagnosed incidentally via routine preoperative tests and subsequent genetic testing. Genetic analysis revealed a heterozygous c.103C>T mutation in the FGA gene—a common variant associated with elevated bleeding risk.

In summary, LUAC concurrent with hereditary dysfibrinogenemia is rare. Based on our experience with this case, we propose the following management insights: (1) Diagnostic evaluation and familial risk stratification in hypofibrinogenemia: For patients with persistent hypofibrinogenemia, detailed family history inquiry and pedigree analysis are critical for diagnosing CD and enabling early intervention. Family members should undergo coagulation function and genetic testing to guide prevention of bleeding/thrombotic events. (2) Perioperative management of LUAC with CD: According to the recommendations in *How I Treat Dysfibrinogenemia* [17], for patients with CD undergoing elective major surgery (e.g., orthopedic, abdominal, gynecological, etc.), it is recommended that the perioperative peak fibrinogen level be > 1.5 g/L. Postoperatively, close monitoring of chest tube output color and volume is required. Given the elevated thrombotic risk, we recommend prioritizing physical thromboprophylaxis, prophylactic anticoagulants are not advised initially due to bleeding concerns. (3) Dynamic monitoring of coagulation function and long‐term follow‐up are essential: Most patients with CD remain asymptomatic clinically, yet they still face a significantly elevated risk of bleeding and thrombotic events. It is advisable to enhance patient education on relevant knowledge, fully inform them of the risks associated with bleeding and thrombosis, schedule regular follow‐ups at hematology outpatient clinics, and initiate early intervention when necessary.

## Author Contributions

**He Zheng:** conceptualization, data curation, formal analysis, methodology, project administration, writing – original draft, writing – review and editing. **Zhengqiang Wan:** formal analysis, project administration, writing – review and editing. **Mingpu Wang, Shu Luo, and Yuzhen Ma:** conceptualization, supervision, writing – review and editing.

## Funding

This study was funded by the “Suining Health Science and Technology Project (24CJDFB38)”.

## Ethics Statement

This study was approved by the Ethics Committee of Suining First People's Hospital (ethics approval number: SNYY‐202507). The patient has signed an informed consent form and agreed to the use of her medical records for the clinical study.

## Consent

The authors have nothing to report.

## Conflicts of Interest

The authors declare no conflicts of interest.

## Data Availability

Data sharing is not applicable to this article as no datasets were generated or analyzed during the current study.
